# Exosomal miR-222-3p derived from dermal papilla cells inhibits melanogenesis in melanocytes by targeting SOX10 in rabbits

**DOI:** 10.5713/ab.24.0182

**Published:** 2024-08-26

**Authors:** Yang Chen, Tingting Lu, Yingying Dai, Yu Xue, Bohao Zhao, Xinsheng Wu

**Affiliations:** 1 College of Animal Science and Technology, Yangzhou University, Yangzhou, Jiangsu 225009, China

**Keywords:** Dermal Papilla Cells, Exosome, Melanocytes, Melanogenesis, miR-222-3p

## Abstract

**Objective:**

Dermal papilla cells (DPCs) play a pivotal role in hair follicle development and can modulate melanogenesis in melanocytes (MCs) through their microenvironment. Our previous studies have demonstrated that the levels of exosomal miR-222-3p derived from DPCs of white Rex rabbits are significantly higher than those of black Rex rabbits. However, the specific role and underlying molecular mechanisms of exosomal miR-222-3p in melanogenesis remain elusive.

**Methods:**

DPCs and MCs were isolated from hair follicles of Rex rabbits and identified using western blotting (WB) and immunofluorescent staining. Exosomes derived from DPCs (DPCs-exos) were characterized using nanoparticle tracking analysis, transmission electron microscopy, and WB. To investigate cell-cell crosstalk mediated by exosomes, MCs were co-cultured with CM-Dil-labeled DPCs-exos. The expression of miR-222-3p in skin tissue and exosomes was quantitatively assessed using quantitative real-time polymerase chain reaction. The transmission of DPCs-secreted exosomal miR-222-3p to MCs was demonstrated using Cy3-labeled miR-222-3p in conjunction with transwell assays. The impact of miR-222-3p on melanin synthesis was evaluated using the NaOH method, cell counting kit-8, and annexin V-fluorescein isothiocyanate/propidium iodide assays. Sex determining region Y-box 10 (SOX10), a potential target gene regulated by miR-222-3p, was validated using a dual-luciferase reporter assay, site-specific mutation, and WB.

**Results:**

Increased levels of miR-222-3p were observed in the skin and DPCs-exos of white Rex rabbits compared to those of black Rex rabbits. Effective internalization of CM-Dil-labeled DPCs-exos by MCs was observed. Furthermore, exosomal miR-222-3p derived from DPCs was transferred to MCs. Functionally, miR-222-3p significantly inhibited MCs proliferation, induced apoptosis and inhibited melanin synthesis. SOX10 was confirmed as a direct target of miR-222-3p in this regulatory cascade.

**Conclusion:**

The findings demonstrate that exosomal miR-222-3p, derived from DPCs, suppresses melanogenesis in MCs by targeting SOX10, thus unveiling a novel mechanism of exosome involvement in melanogenesis.

## INTRODUCTION

Melanogenesis in melanocytes (MCs) determines the color of fur and skin in mammals [1,2]. The dermal papilla cells (DPCs) act as the signaling center of the hair follicle growth cycle, thereby generating a microenvironment that can potentially influence the function of MCs [3]. Melanogenesis in MCs is synchronized with the hair cycle; MCs proliferate and differentiate during the anagen phase and undergo apoptosis during catagen. In addition, the interaction between the bulbar MCs and dermal papillae (DP) is evident from their anatomical location, where the matrix and DP are separated by a very thin and permeable basal layer membrane [4]. Therefore, MCs are often subject to negligible interaction of the DP in hair follicles.

Exosomes (Exos), key components of extracellular vesicles, facilitate the transfer of various biomolecules from the donor cells to recipient cells, mediating cell-to-cell communication [5,6]. Several studies have demonstrated that exosomes from dermal papillae (DPC-exos), dermal fibroblasts, and mesenchymal stem cells can effectively activate hair follicle stem cell proliferation, thereby promoting hair growth [7–11]. In addition, low-generation DPCs-exos can stimulate hair growth by promoting the proliferation of outer root sheath and hair matrix cells [12]. DPCs-exos can increase the hair-inductive capacity of the cultured dermal papilla spheres to promote hair growth and regeneration [13]. These studies indicate that DPCs-exos can play a key role in hair follicle regeneration [14]. However, the specific role of DPCs-exos in regulating melanogenesis in MCs remains unclear.

Previous studies have demonstrated the involvement of exosomes in melanogenesis, particularly in epidermal keratinocytes. For example, RAB1B influences melanin transport between MCs and keratinocytes through coupled exo/endocytosis [15]. Keratinocyte derived miR-330-5p can significantly reduce melanin synthesis and tyrosinase (TYR) expression in MCs [16]. Moreover, over 30 differentially expressed miRNAs, including exosomal miR-203 and miR-3196, which fine-tune MC pigmentation, were identified in exosomes from Caucasian and Black keratinocytes [17]. Therefore, exosomes play a crucial role in modulating melanin deposition by selectively transporting specific miRNAs to MCs, altering target gene expression, and regulating pigmentation enzyme activity [18,19]. Regarding miR-222-3p, it has been implicated in melanoma [20–22] and has been identified in extracellular vesicles [23]. Previous studies have shown that miR-222-3p plays a role in the formation of Rex Rabbits’ fur color [24]. Additionally, miR-222-3p expression was found to be significantly higher in the skin of white Rex rabbits than in black Rex rabbits. However, the potential effect of DPCs-exos miR-222-3p on melanogenesis in MCs remains to be explored.

This study revealed significantly higher levels of miR-222-3p in the skin and DPCs-derived exosomes of white Rex rabbits compared to black ones. We analyzed the transfer of miR-222-3p from DPCs to MCs, its targeting relationship with sex determining region Y-box 10 (SOX10), and its impact on melanin production. The findings suggest a novel regulatory role for exosomal miR-222-3p in melanogenesis, potentially advancing our understanding of melanin metabolism and skin/hair coloration.

## MATERIALS AND METHODS

### Cell isolation and culture

The animal experiments were approved by the Animal Ethics Committee of Yangzhou University, China (approval No. 202103283) and were strictly implemented according to the Guide for the Care and Use of Laboratory Animals.

Skin samples from the backs of Rex rabbits were collected and immersed in phosphate buffered saline (PBS) containing 3% cyanine/streptomycin. These tissues were cut into strips measuring 0.25 cm×0.5 cm and digested overnight at 4°C in 0.25% dispase II (Gibco, Grand Island, NY, USA). The epidermis and dermis were then separated, retaining only the dermal tissue. To isolate and culture DPCs, the dermis was minced and digested with 1 mg/mL collagenase (Gibco, USA) at 37°C for 1 h. The digest was then filtered through a 70 μm mesh and centrifuged to collect the cells. DPCs were cultured with mesenchymal stem cell medium (MSCM) (Sciencell, San Diego, CA, USA) at 37°C in 5% (v/v) CO_2_. MCs were isolated as described previously with slight modification [25,26]. Collagenase digestion was conducted after additional dissection of hair follicles from the dermis. After 70 μm and 40 μm filtration, the cells were then collected by centrifugation. MCs were cultured with M254 medium (Gibco, USA) containing HMGS-2 (Gibco, USA) at 37°C in 5% (v/v) CO_2_.

### Antibodies

The following antibodies were used: anti-vimentin (VIM) antibody at 1:800 for western blotting (WB) and 1:200 for immunofluorescent (IF) (BM0135; BOSTER, Wuhan, China), anti-α-smooth muscle actin (SMA) antibody at 1:800 for WB and 1:200 for IF (BM0002; BOSTER, China), anti-SOX2 antibody at 1:250 for IF (66411-1-Ig; Proteintech, Wuhan, China), anti-Versican antibody at 1:250 for IF (DF10007; Affinity, Jiangsu, China), anti-tyrosinase-related protein 2 (TYRP2) antibody at 1:200 for IF (AF5303; Affinity, China), anti-TYR antibody at 1:1,000 for WB and 1:200 for IF (Ab6211; Abcam, Cambridge, MA, USA), anti-microphthalmia-associated transcription factor (MITF) antibody at 1:1,000 for WB and 1:200 for IF (SC515925; Santa Cruz, CA, USA), anti-glyceraldehyde-3-phosphate dehydrogenase (GAPDH) antibody at 1:10,000 for WB (60004-1-Ig; Proteintech, China), anti-CD9 antigen (CD9) antibody at 1:5,000 for WB (60232-1-Ig; Proteintech, China), anti-tumor susceptibility gene 101 (PTSG101) antibody at 1:5,000 for WB (28283-1-AP; Proteintech, China), anti-SOX10 antibody at 1:1,000 for WB (66786-1-Ig; Proteintech, China), HRP-conjugated Affinipure Goat Anti-Mouse IgG(H+L) at 1:10,000 for WB, HRP-conjugated Affinipure Goat Anti-Rabbit IgG(H+L) at 1:10,000 for WB and 1:200 for IF (SA 00001-2; Proteintech, China), Cy3-conjugated Affinipure Goat Anti-Mouse IgG(H+L) at 1:2,000 for IF (SA00009-1; Proteintech, China) and Cy3-conjugated Affinipure Goat Anti-Rabbit IgG(H+L) at 1:2,000 for IF (SA00009-2; Proteintech, China).

### Western blot analysis

Samples were lysed with a solution containing PMSF and centrifuged at 13,000 g at 4°C for 5 min to collect the supernatants. The protein concentration was determined using the BCA kit (Beyotime, Shanghai, China). After denaturation, the protein was transferred to the PVDF membrane by sodium dodecyl sulfate-polyacrylamide gel electrophoresis using SurePAGE Bis-Tris 10×8, 4% to 20% 10 wells (GenScript, Jiangsu, China). Primary antibodies were diluted according to the manufacturer’s instructions and incubated at 4°C overnight. The second antibody was incubated at room temperature for 1 to 2 h. After ECL hypersensitive luminescence processing, the images were collected.

### Immunofluorescent staining

When cells reached 50% confluency, they were fixed with 4% paraformaldehyde for 30 min. Triton X-100 (Solarbio, Beijing, China) was used for permeabilization at 37°C for 20 to 30 min. The cells were blocked with 1% bovine serum albumin (BSA) for 1 h. Cells were incubated with the primary antibody at 4°C for overnight and with a Cy3-labeled secondary antibody at room temperature for 1 to 2 h. After 4’,6-diamidino-2-phenylindole (DAPI) staining, images were captured under an inverted fluorescence microscope (IX71; Olympus, Tokyo, Japan).

### L-3,4-dihydroxyphenylalanine staining

When the confluency reached to approximately 80% to 90%, MCs were fixed with 4% paraformaldehyde at room temperature for 20 min. Subsequently, the cells were incubated with 0.1% L-3,4-dihydroxyphenylalanine (L-DOPA) (Sigma, China) dissolved in 1% Triton X-100 (Solarbio, China) at 37°C for 3 h. The images were captured under a microscope (IX71; Olympus, Japan).

### Melanin contents analysis

A solution of 1 mol/L NaOH solution containing 10% dimethyl sulfoxide (DMSO) was added to the samples and incubated at 80°C for 1 h. The samples were then added to 96-well plates (100 μL/wells), and three parallel triplicates were used for each sample. The absorbance was measured at 405 nm by a microplate reader (Infinite M200 PRO; TECAN, Männedorf, Switzerland). The relative melanin content was determined using the formula: % = (test_A405_ – blank_A405_)/(control_A405_ – blank_A405_)×100%.

### Exosome isolation, labeling, and identification

DPC culture medium was collected and centrifuged to obtain the supernatant. An ultrafiltration tube was used to concentrate the cell supernatant. Thereafter, half the volume of total exosome isolation reagent (from cell culture media) (ThermoFisher, Waltham, MA, USA) was added, mixed thoroughly, and incubated at 2°C to 8°C overnight. Centrifugation was performed at 10,000 g at 4°C for 1 h. The pellet was collected, resuspended in PBS, and identified as exosomes from DPCs.

The membrane dye CM-Dil (Beyotime, China) was added and incubated in the dark for 20 min. PBS was added and centrifuged at 3,000 g at 4°C for 10 min. The supernatant was then collected, and exosomes were extracted again according to the exosome extraction procedure. Then, 20 μL exosomes were dropped into the electron microscope copper mesh grid and retained for a while (>1 min). After negative staining with uranyl acetate solution for 10 min, the samples were dried at room temperature. They were then observed and photographed under a biological transmission electron microscope (HT-7700; Hitachi, Tokyo, Japan).

After that, based on nanoparticle tracking analysis (NTA), the particle size of the extracted exosomes was analyzed. The sample pool of the ZetaView nanoparticle tracking analyzer was cleaned with deionized water and calibrated with polystyrene microspheres (100 nm). After the sample pool had been cleaned with PBS, exosomes were detected.

### Detection of Cy3-labelled miR-222-3p

Transwell chambers (Corning, New York, USA) were utilized to establish a co-culture system. DPCs were seeded into the upper chambers, while MCs were seeded into the lower culture plates for 24 h. Following the transfection of DPCs with Cy3-miR-222-3p mimics (NC), the transwell chamber was positioned over the culture plate containing fresh medium. The red fluorescence of Cy3 in MCs was observed after 48 h culture. Subsequently, the transwell chamber was removed, and MCs were collected for IF staining using TYR antibody.

### RNA extraction and quantitative real-time polymerase chain reaction

Total RNA was extracted from the tissues, cells, and exosomes using an RNAsimple total RNA extraction kit (TIANGEN, China). RNA was reverse transcribed into cDNA using HiScript III qRT SuperMix (Vazyme, China). Quantitative real-time polymerase chain reaction (RT-qPCR) was then performed according to the instructions of ChamQ SYBR qPCR Master Mix (Vazyme, China), with GAPDH as the internal reference. The RNA was reverse-transcribed into miRNA cDNA based on miRcute Plus miRNA First-Strand cDNA kit (TIANGEN, China). miRNA expression was detected using the miRcute Plus miRNA qPCR kit (SYBR Green) (TIANGEN, China), with small nuclear RNA U6 (U6) as the internal reference. The information about primers used is shown in Table 1.

### Cell proliferation assay

MCs were collected 24 h after the transfection and inoculated into 96-well plates (100 μL/well) to culture for 0 h, 24 h, 48 h, 72 h. Thereafter, a cell counting kit-8 (CCK-8) kit (Vazyme, China) was used, and a CCK-8 solution (10 μL/well) was added to the cells. Each sample was assayed in triplicate. The absorbance at 450 nm was measured by a microplate reader (Infinite M200 PRO; TECAN, Switzerland) after incubation for 4 h to record the cell proliferation.

### Cell apoptosis determination

The samples were treated with Annexin V-FITC apoptosis detection kit (Vazyme, China). Annexin V-FITC and PI staining solution were added and incubated at the room temperature in the dark. Apoptosis rates were analyzed using flow cytometry (FACSAria SORP; Becton Dickinson, Franklin Lakes, NJ, USA), with each sample assayed in triplicate. The apoptosis rate was calculated by the sum of the percentage of Q2 and Q3 quadrants.

### Luciferase reporter assay

SOX10 3′-UTR target fragment (WT) and mutant fragment (MUT), with TGTAGC mutated to ACATCG, were inserted into the pmirGLO vector at *Sac*I and *Xho*I restriction sites. The miR-222-3p mimics/inhibitor (NC), WT/MUT, and pRL-TK vector were co-transfected in RAB-9 (ATCC CRL-1414, Rockville, MD, USA). The luciferase activity was detected using a dual luciferase reporter assay kit (Vazyme, China).

### Statistical analysis

The data of RT-qPCR was processed by the 2^−ΔΔCt^ method [27,28]. Statistical analyses were performed using the t-test and one-way analysis of variance. * Indicates significantly different (p<0.05), ** indicates extremely significant difference (p<0.01). The results have been presented as mean± standard deviation. GraphPad Prism 8 was used for the plotting.

## RESULTS

### Isolation and identification of dermal papilla cells and melanocytes

**Figure 1:**
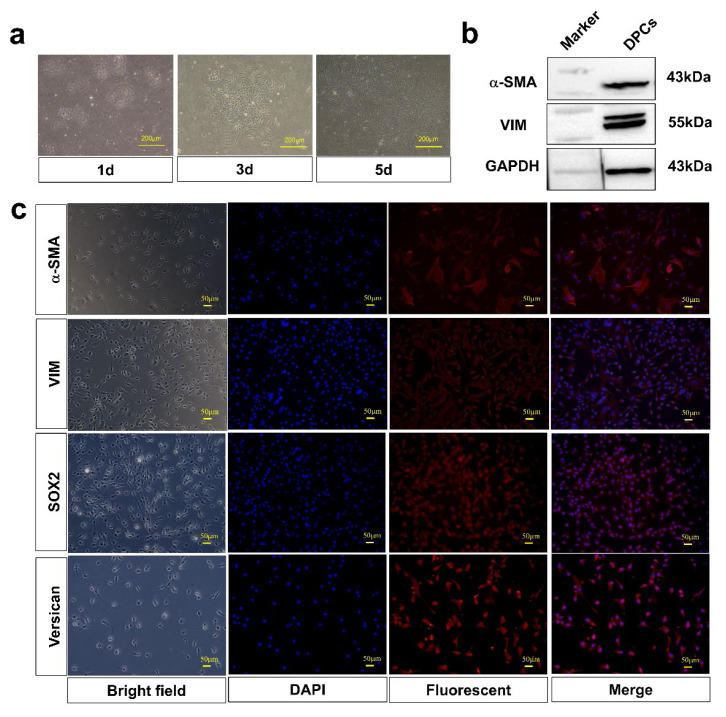
Isolation and identification of dermal papilla cells. (a) Morphological observation of DPCs at 1, 3, and 5 d post-isolation. (b) Western blot analysis to detect the expression of DPC-specific markers α-SMA and VIM. (c) Immunofluorescence staining of DPCs using antibodies against α-SMA, VIM, SOX2, and Versican. DPCs, dermal papilla cells; α-SMA, alpha smooth muscle actin; VIM, vimentin; SOX2, sex determining region Y-box 2.

**Figure 2:**
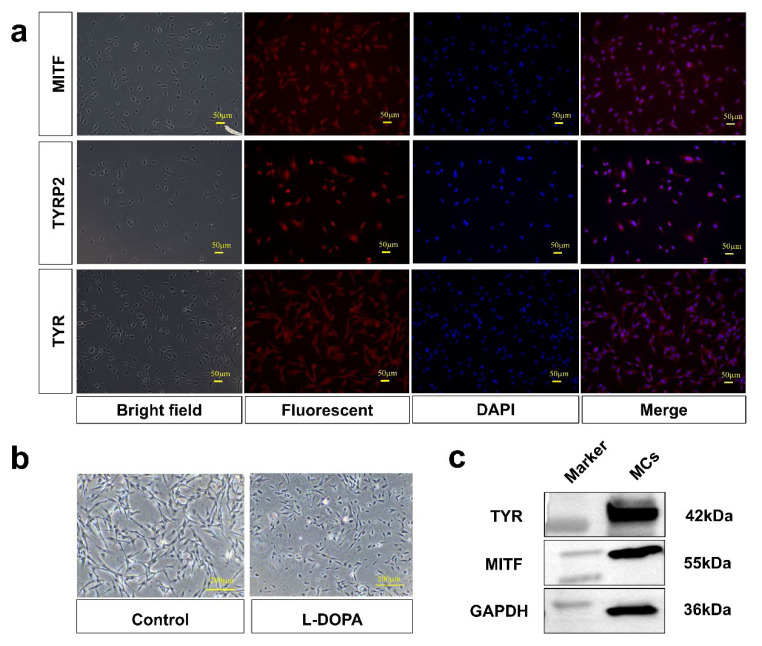
Isolation and identification of melanocytes. (a) Immunofluorescence staining of MC-specific markers MITF, TYRP2, and TYR. (b) MC identification through L-DOPA reactivity. (c) Western blot analysis of TYR and MITF proteins in MCs. MC, melanocytes; MITF, microphthalmia-associated transcription factor; TYRP2, tyrosinase-related protein 2; TYR, tyrosinase, L-DOPA, L-3,4-dihydroxyphenylalanine.

After isolation for 24 h, primary DPCs displayed aggregative towards outwards and mostly showed long spindle-shaped or irregular shapes. After 3 days, the cells showed radial growth, but after 5 days, the cells exhibited agglutination (Figure 1a). The α-SMA and VIM proteins were identified by WB and IF staining in DPCs (Figure 2b and 2c). Additionally, the isolated MCs showed the bipolar or tripolar morphology. The MCs-specific markers MITF, TYR, and TYRP2 were then identified by IF staining (Figure 2a). Black particles were observed in MCs following L-DOPA staining (Figure 2b). MITF and TYR proteins were detected by WB analysis (Figure 2c). These results indicated that the isolated cells were DPCs and MCs, respectively.

### Internalization of DPCs-exosomes to melanocytes

**Figure 3:**
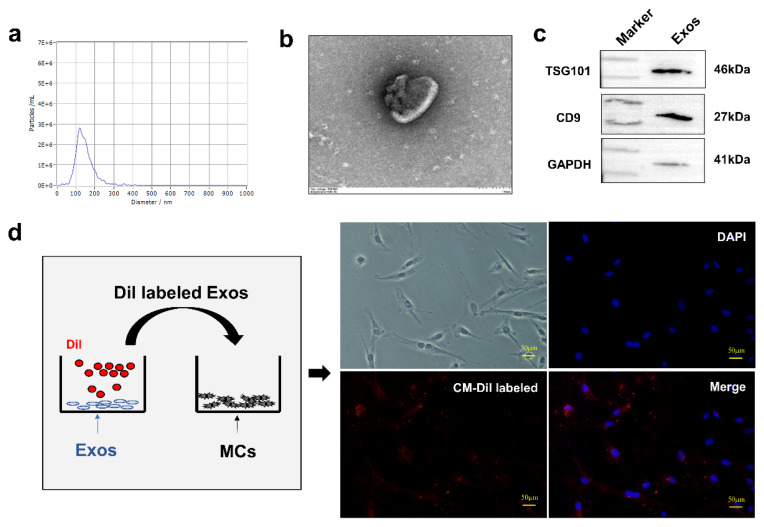
Identification of DPCs-exosomes. (a) Determination of DPCs-exosome diameters through nanoparticle tracking analysis. (b) Visualization of DPCs-exosome morphology by transmission electron microscopy. (c) Western blot detection of exosome markers CD9 and TSG101 in DPCs-derived exosomes. (d) Labeling and internalization of DPCs-exosomes by MCs using CM-Dil. DAPI staining indicates the nucleus. DPCs, dermal papilla cells; CD9, CD9 antigen; TSG101, tumor susceptibility gene 101; DAPI, 4′,6-diamidino-2-phenylindole.

Exosomes were isolated from DPC supernatants. NTA showed that the DPCs-exos diameter was around 120 nm, with both in the range of 30 nm to 150 nm (Figure 3a). The structures of exosomes appeared as saucer or concave hemispheres by TEM (Figure 3b). Exosome-specific protein CD9 and TSG101 were also detected in exosomes by WB (Figure 3c). So, the isolated materials were confirmed to be exosomes. To confirm the adsorption of DPCs-derived exosomes by MCs, CM-Dil labeled exosomes were added to MCs. CM-Dil-labeled exosomes were primarily observed to cluster within MCs (Figure 3d). This suggested the effective internalization of CM-Dil-labeled DPCs-exos by MCs.

### miR-222-3p is enriched in the skin and DPCs-exosomes of white Rex rabbits and transferred to melanocytes

**Figure 4:**
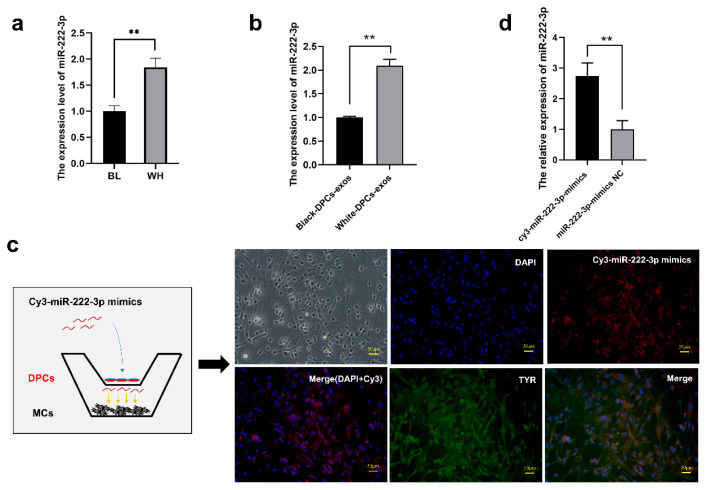
Transfer of miR-222-3p derived from DPCs-exosomes to melanocytes. (a) Expression profile of miR-222-3p in the skin of white and black Rex rabbits (BL, Black; WH, White). (b) Quantification of miR-222-3p expression levels in DPCs-derived exosomes from white and black Rex rabbits by RT-qPCR. (c) Illustration of cell co-culture system in a transwell chamber, demonstrating the transfer of Cy3-labeled miR-222-3p mimics to MCs (red fluorescence). TYR immunofluorescence staining indicates MCs, while DAPI staining indicates the nucleus. (d) Relative expression level of miR-222-3p in the transwell chamber, with NC as the control. DPCs, dermal papilla cells; RT-qPCR, quantitative real-time polymerase chain reaction; MC, melanocytes; DAPI, 4′,6-diamidino-2-phenylindole; NC, negative control. ** Indicates significant differences among groups (p<0.01).

The mRNA level of miR-222-3p in the skin and DPCs-exos of white and black Rex rabbits was detected by RT-qPCR. The expression of miR-222-3p was found to be higher in the skin and DPCs-exos of white Rex rabbits (p<0.01) (Figures 4a and 4b). We reasoned that miR-222-3p plays a key role in differences observed in MCs melanin synthesis caused by DPCs-exos. Furthermore, Cy3-labeled miR-222-3p mimics were transfected into DPCs, and DPCs, as well as MCs, were co-cultured in a Transwell chamber. We observed a clear red fluorescence in MCs (Figure 4c). In addition, the expression of miR-222-3p was significantly increased in comparison with NC (p<0.01) (Figure 4d). It was demonstrated that DPCs could secrete the exosomal miR-222-3p into MCs.

### miR-222-3p inhibited MC proliferation and melanin synthesis

**Figure 5:**
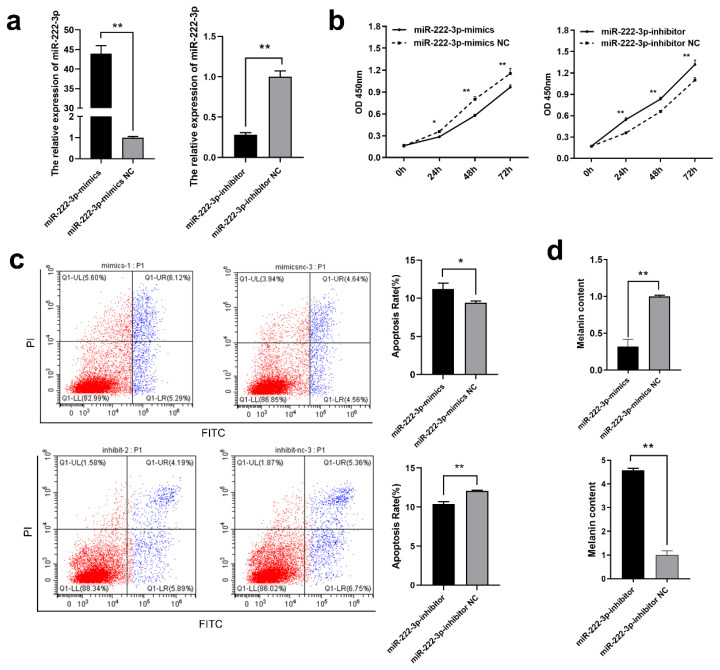
miR-222-3p inhibits melanin synthesis in melanocytes. (a) Quantification of miR-222-3p expression in MCs following transfection with mimics and inhibitor using RT-qPCR. (b) Analysis of miR-222-3p on MC proliferation at 0 h, 24 h, 48 h, and 72 h using the CCK-8 assay. (c) Evaluation of miR-222-3p on MC apoptosis through Annexin V-FITC/PI flow cytometry. (d) Examination of miR-222-3p on melanin synthesis in MCs using the NaOH method. RT-qPCR, quantitative real-time polymerase chain reaction; CCK-8, cell counting kit-8; MC, melanocyte; V-FITC/PI, V-fluorescein isothiocyanate/propidium iodide. ** Indicates p<0.01 and * indicates p<0.05.

Next, we analyzed the effect of miR-222-3p on MCs proliferation, apoptosis, and melanin synthesis. Using CCK-8 assay and annexin V-fluorescein isothiocyanate/propidium iodide (V-FITC/PI) flow cytometry, it was found that miR-222-3p mimics significantly inhibited MCs proliferation and promote apoptosis (p<0.05), whereas miR-222-3p inhibitor exhibited an opposite effect (p<0.01) (Figure 5a–5c). It was suggested that miR-222-3p was positively correlated with MCs apoptosis. Further, it was found that after the transfection with miR-222-3p mimics, melanin content was significantly decreased (p< 0.01), whereas melanin content was significantly increased with transfection with miR-222-3p inhibitor (p<0.01) (Figure 5d). Thus, after miR-222-3p enters into MCs, its functional pathway needs further exploration.

### SOX10 is a direct target of miR-222-3p

**Figure 6:**
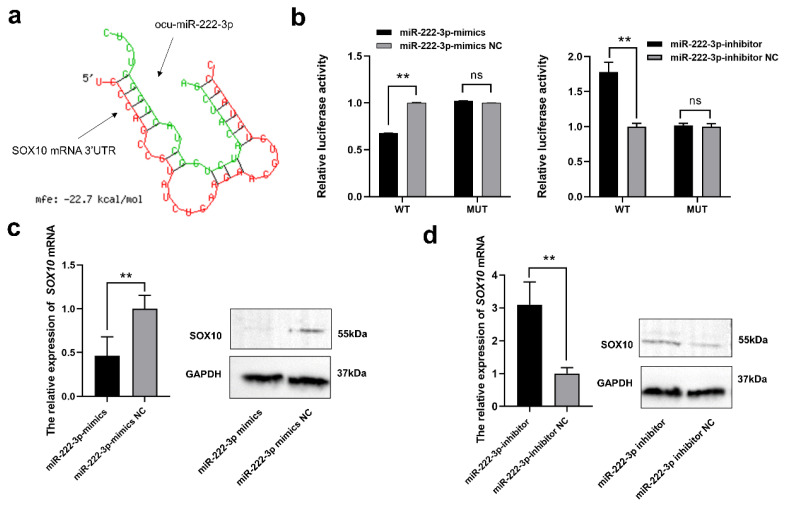
Regulation of miR-222-3p targeting SOX10. (a) Prediction of miR-222-3p targeting SOX10 using RNAhybrid. (b) Verification of miR-222-3p and SOX10 interaction through dual luciferase system and site-specific mutation techniques. (c) Analysis of SOX10 mRNA and protein expression after miR-222-3p mimics transfection in MCs by RT-qPCR and WB. (d) Analysis of SOX10 mRNA and protein expression after miR-222-3p inhibitor transfection in MCs by RT-qPCR and WB. SOX10, sex determining region Y-box 10; RT-qPCR, quantitative real-time polymerase chain reaction; MCs, melanocytes; WB, western blotting. ** Indicates significant differences among groups (p<0.01).

**Figure 7:**
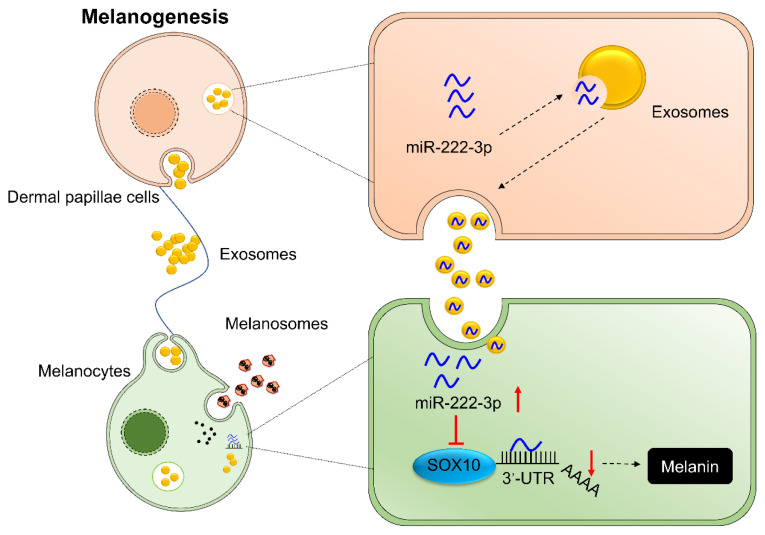
Exosomal miR-222-3p derived from dermal papilla cells inhibits melanocytes melanogenesis by targeting SOX10.

The mature ocu-miR-222-3p sequence AGCUACAUCUG GCUACUGGGUCUC was obtained from miRBase and NCBI databases. The binding sites between ocu-miR-222-3p and SOX10 were then predicted using RNAhybrid (Figure 6a). The regulatory relationship between miR-222-3p and SOX10 was further analyzed by using a dual luciferase system and site-specific mutation techniques. When co-transfected with SOX10-3′UTR-WT and miR-222-3p mimics, the luciferase relative activity decreased markedly (p<0.01), but no significant effect on activity was observed when SOX10-3′UTR-MUT and miR-222-3p mimics were co-transfected (p>0.05). In contrast, the luciferase activity was significantly increased after co-transfection with SOX10-3′UTR-WT and miR-222-3p inhibitor (p<0.01) (Figure 6b). Furthermore, miR-222-3p mimics were transfected in MCs, resulting in a significant decrease in MCs at the RNA and protein levels (Figure 6c and 6d). In summary, after entering into the MCs, DPCs-derived exosomal miR-222-3p were able to inhibit melanin synthesis in MCs by targeting SOX10 (Figure 7).

## DISCUSSION

In addition to its known ability to induce hair follicle growth, dermal papilla serves as a significant pigment regulator within hair follicles. DPC stem cell factors can bind to receptor c-KIT present on MCs to regulate melanin synthesis [29,30]. Endothelin 3, a marker gene for DPCs, has been reported to induce differentiation and proliferation of MCs and to modulate skin pigmentation in transgenic mice [31–33]. Moreover, human DPCs secrete different MC chelators, and it is hypothesized that these molecules can effectively cause MCs to migrate to hair follicles [34]. Regarding melanogenesis, it has been reported that DPCs can promote pigmentation by affecting Agouti protein [35,36]. The Extracellular matrix of DPCs has also been documented to stimulate the activity of TYR, a rate-limiting enzyme for melanin synthesis [37]. In addition, previous reports have confirmed that DPCs can affect melanogenesis in MCs, but the transfer factor involved in this process remains unknown. In this study, DPCS and MCs were successfully isolated and identified by WB and IF staining, laying the foundation for following investigations into cell-cell crosstalk and its role in melanin regulation.

Previous research has established that exosomes can carry miRNAs, facilitating cellular communication [5,6]. Additionally, several sources of exosome miRNAs have been found to affect the process of melanogenesis, such as keratinocytes-derived exosomal miR-330-5p and miR-200c [38,39], amniotic stem cells-derived exosomal miR-181a-5p and miR-199a [40], milk-derived exosomal miR-2478 [41], and so on. Interestingly, previous studies have conducted miRNA profiling of MC lines, melanocytoma lines, and their tissue samples and found the presence of miR-222-3p in all of them [42]. It has been reported that miR-222-3p can function as the downstream intercept of curcumin’s inhibition of melanoma growth [21,22]. In this study, we found that the expression level of miR-222-3p in skin and DCPs-exos of white Rex rabbits was significantly higher than that of black Rex rabbits. Notably, DPCs-exos can carry miR-22-3p into MCs. Further experiments showed that miR-222-3p inhibited proliferation of MCs, promoted their apoptosis and suppressed melanin synthesis.

In order to gain a deeper understanding of the miR-222-3p regulation mechanism in melanogenesis within MCs, SOX10 was postulated as a potential target gene for miR-222-3p. SOX10 is a specific marker of MCs derived from the neural crest cells [43,44] and plays a pivotal role in regulating MC proliferation [45–47]. As an essential molecule for melanin synthesis, SOX10-knocked-out mice have been found to lose most or all of their MCs [48]. We verified the targeting relationship between miR-222-3p and SOX10 through luciferase reporter assay. Moreover, miR-222-3p attenuated the SOX10 mRNA and protein expression, modulating the proliferation and apoptosis of MCs. In summary, miR-222-3p derived from DPCs-exos can inhibit MC proliferation, promote apoptosis, and inhibit melanogenesis by targeting SOX10. This reveals the specific role and underlying molecular mechanisms of exosomal miR-222-3p in melanogenesis. These findings pave the path for development of novel strategies related to melanin metabolism and skin/hair coloration.

## Figures and Tables

**Table 1:** The sequences of primers used for quantitative real-time polymerase chain reaction

Name	Sequence
miR-222-3p	GCTACATCTGGCTACTGGGTCTC
U6	CAAGGATGACACGCAAATTCG
SOX10	F: CAAGCTTTGGAGGCTGCTGA
	R: GGGCTGCCTTCCCATTCTTC
GAPDH	F: CACCAGGGCTGCTTTTAACTCT
	R: CTTCCCGTTCTCAGCCTTGACC

## Data Availability

All data obtained or analyzed in this study are available from the corresponding author upon request.
